# On the effectiveness of Buddhist view of life and death in regulating mortality salience

**DOI:** 10.3389/fpsyg.2024.1523125

**Published:** 2025-01-08

**Authors:** Jiaqing Qiu, Xianjun Xu

**Affiliations:** Department of Psychology, School of Humanities, The University of Tongji, Shanghai, China

**Keywords:** mortality salience, Buddhist, view of life and death, reincarnation, Yogacara Buddhism

## Introduction

Death is an inevitable fate for human beings. Awareness of death diminish personal sense of security, increase fear (Alkozei et al., 2019), and trigger death anxiety (Lehto and Stein, 2009). Death anxiety profoundly impacts mental health and is a potential cause of mental disorders, significantly contributing to their persistence and recurrence (Menzies et al., 2018). Thus, alleviating death anxiety is crucial for enhancing quality of life.

Terror Management Theory (TMT), built upon Becker's ideas (Greenberg et al., 1997) and refined by Greenberg, addresses the emotions of fear and anxiety induced by death (Mikulincer et al., 2003). According to it, death possesses two inherent characteristics: inevitability and unpredictability (Goldenberg et al., 2008). The inevitability of death makes individuals aware of their “certain end,” generating endless fear and anxiety (Fritsche et al., 2008). Its unpredictability fosters a sense of crisis and urgency, as individuals realize that death is beyond their control. So all human behaviors are, in a sense, efforts to resolve existential contradiction between life and death (Freud, 1981). The pursuit of survival and the escape from the inevitability of death are lifelong issues.

Mortality Salience (MS), a core concept of TMT, directly addresses death anxiety. When individuals become aware of their mortality, death anxiety latent in daily life triggers MS which increases motivation to accept cultural anxiety buffer (Pyszczynski et al., 2015). Becker (1973) emphasizes that recognition of cultural value significantly impacts death anxiety. In the absence of cultural identification, individuals often feel more anxious. The enhancement of cultural worldview has a significant effect on alleviating anxiety (Pandya and Kathuria, 2021), with self-esteem maintenance and intimate relationships also considered to be important factors influencing MS (Hart et al., 2005).

Cultural worldviews shape individuals' perceptions of death and alter conditions under which MS operates (Neimeyer et al., 2011), affecting the degree of death anxiety. For example, natural sciences view death as a natural process, Christians sees it as a gateway to another world, and Buddhists holds a perspective of reincarnation (Cheung and Ho, 2004). As Christians and Buddhists do not view death as the end of the world for individuals, those believed in them experience significantly less death anxiety than those in the first culture (Mohammadzadeh and Najafi, 2020; Lewis, 2014). Moreover, strong religious faith or belief in an afterlife can reduce death anxiety (Alvarado et al., 1995; Pandya and Kathuria, 2021). Cultural beliefs give meaning and purpose, helping individuals face death anxiety effectively as cultural anxiety buffers (Schindler et al., 2023).

Buddhism, a traditional culture of South Asia, will be explored in this paper to illustrate how its view on life and death could function as an anxiety buffer, and help individuals overcome death anxiety to maintain mental health.

### The continuity of life and death

Buddhism teaches that all living beings are a continuity of life and death. If one believes that death marks the absolute end, this is a kind of “nihilism” that Buddhism opposes. According to Buddhism, when life ends, it will leave imprints, i.e., retributive consequence produced by Karma. The previous life is the cause, and the present life is the effect; the present life is the cause, and the future life is the effect. The chain of life and death is endless, with neither beginning nor end. “Life and death coexist, like the two ends of a scale, rising and falling equally” (Maitreya, 2008). This teaching significantly reduces the crisis and urgency brought by uncertainty, enhances the sense of control, and mitigates death anxiety (Fritsche et al., 2008).

When individuals face reminders of death, they feel the inevitability and unpredictability of death and seek a sense of existence, value, and meaning (Goldenberg et al., 2008). The Buddhist view on continuity of life and death points out that the accumulated Karma of past lives shapes the present, and present actions shape the future (Guoliang, 2000). In other words, the inevitability of death does not render present life meaningless. Individuals should calmly accept death and strive for happiness in future lives. The concept of continuity provides a path for individuals to understand and cope with the trauma of death, imbuing current life with a sense of continued value and meaning, allowing a more composed approach to the heavy topic about death.

In fact, almost all cultures consider death a taboo (Feifel, 1959; Crespo-Fernández, 2023). The explanations of simultaneity and continuity of life and death allow individuals to routinely talk about this heavy topic, providing an entry for discussing life and death. According to psychoanalysis, talking about death inherently alleviates death anxiety repressed in the unconscious (Freud, 1920). Furthermore, Yen (2013)'s research on attitudes toward death indicates that Asians can alleviate death anxiety through beliefs in fatalism and Karma. The continuity of life and death is an expression of fatalism: both life and death are inevitable (Paranjpe, 1984). This view reduces the pressure on individuals in their current lives. Death is not the end forever but a new beginning. Wishes unfulfilled in this life can be pursued in the next.

### No life, no death

The four noble truths of Buddhism focus on the existence of suffering, cause of suffering, cessation of suffering, and path to liberation from suffering. These truths provide a systematic view on death, indicating the source of death anxiety, the way to manage it, and the state of complete releasing from it (Mun-Keat, 2000).

The truth of suffering refers to the existence of pain, dissatisfaction, and unhappiness in life. Life is fraught with eight types of suffering: the suffering of birth, aging, illness, death, being unable to obtain what one desires, association with the hated, separation from the loved, and the flourishing of the five aggregates. Death is one of them. The truth of the cause of suffering explains that the root of suffering, including death, lies in “self-attachment.” It suggests that the origin of all suffering is the attachment to a permanent and controlling self. This attachment generates intense fear and reluctance when approaching death, leading to a cycle of rebirth and new death anxiety (Mun-Keat, 2000).

The truth of the cessation of suffering discusses the end of suffering. It states that by eliminating attachment and craving for the self, one can escape the cycle of rebirth and attain Nirvana. Nirvana is not a passive state of nothingness, but a transcendent state beyond suffering and life, representing the ultimate freedom and liberation sought in Theravada Buddhism. The truth of the path outlines the eightfold path as the means to end suffering, comprising Right View, Right Intention, Right Speech, Right Action, Right Livelihood, Right Effort, Right Mindfulness, and Right Concentration (Mun-Keat, 2000). Many studies have confirmed that mindfulness practice has positive effects on people's body and mind (Black and Slavich, 2016; Schutte and Malouff, 2014; Azam et al., 2015; Chiesa et al., 2011; Hofmann et al., 2010). The truth of the path serves as a guide for spiritual practice, suggesting that by following the eightfold path, individuals can achieve the cessation of suffering and ultimate Nirvana.

All living beings are emotional creatures, referred to in Buddhism as sentient beings, experience suffering, including death anxiety. While explaining the origins of death anxiety, the four noble truths also provide a method to manage it, proposing that through the practice of the path, individuals can reach a state free from death anxiety. This state entails not identifying the body, mind, or any unity as the self, thus eliminating attachment and craving, and attaining true freedom. William et al. has pointed out that death anxiety can be alleviated by overcoming their attachment to the body, which is one part of the self-attachment (Van Gordon et al., 2018).

With life comes death, but no life, no death. This idea offers a systematic explanation of the process of death anxiety, helping individuals confront this obscure theme. So individuals can better understand and cope with death anxiety.

However, the truth of suffering implies the idea of escaping from real life, and Zen can be an effective supplement. Zen advocates an outlook on real life and does not require such forms as sitting in meditation, reading scriptures, or worshiping the Buddha; one can become a Buddha in daily life. Zen emphasizes a “joy of life” rather than a “suffering of life,” which is very suitable for modern people (Yun, 2009).

### There is no life or death, only the Alaya-vijnana

Individuals often perceive life as linear, with a defined beginning and an inevitable end marked by death (Northrup, 2002). This linear view of time leads individuals to see time as a finite and valuable resource that can be quantified, monitored, and managed (Bruce, 2007). However, Yogacara Buddhism offers a different perspective. It denies the reality of life and death, suggesting that both the self and the external world are mere illusions projected by the Alaya-vijnana. “Revolving into sentient beings, and revolving into dharmas” (Lusthaus, 2002).

“You should observe the nature of the dharma realm, and everything is created only by the mind” (CBETA, 2024). This teaching emphasizes the mind as the foundation of reality. More specifically, Yogacara Buddhism divides the mind into eight levels: seeing-vijnana, hearing-vijnana, smelling-vijnana, tasting-vijnana, kinetic-vijnana, vijnana, Manas-vijnana, and Alaya-vijnana. The first five vijnanas are within the scope of human perception, with distinctions depending on specific sensory organs. Vijnana, also known as the sixth consciousness, is the mental faculty (Guoliang, 2000).

Manas-vijnana, the seventh consciousness, continuously operates, mistakenly perceiving the perception of the Alaya-vijnana as a constant self, thus firmly believing in an eternal self. Consequently, it harbors four types of attachment: self-delusion, self-belief, self-arrogance, and self-love. The activities of it are so subtle that man cannot perceive it. The root of death anxiety lies in Manas-vijnana (Guoliang, 2000).

Alaya-vijnana, the eighth consciousness, functions as a repository, storing all seeds of dharma (all phenomena). More specifically, it stores seeds (word representation) produced by the activities of the six vijnanas. Additionally, Alaya-vijnana can project the external world and sentient beings. In other words, all phenomena are manifestations or projections of seeds in Alaya-vijnana (Guoliang, 2000).

Therefore, it is not a soul that traverses around the six realms, but rather the six realms are manifested in Alaya-vijnana. The ever-changing Alaya-vijnana forms a continuous chain of cause and effect. All sentient beings live through boundless time, with each life linked to the next, and their minds and bodies constantly in flux. This continuity results in various afflictions and the creation of Karma, leading to the cycle of rebirth into different states of beings. True liberation can only be achieved through the transformation of vijnanas into wisdom (Xianjun, 2023). While the physical body perishes, an individual's life can perpetually reincarnate through Alaya-vijnana, thus supernatural immortality is achieved through continuity of it (Vail III et al., 2019).

Yogacara Buddhism breaks the linear view of time that necessitates confronting the ultimate topic of life's end (Dechesne et al., 2003), providing a protective barrier of the supernatural myth of eternal life. This offers individuals hope for endless rebirth. At the same time, by discarding linear and temporal thinking, individuals can focus more on living in the present and alleviate death anxiety by cherishing the present moment. This aligns with Gestalt therapy's emphasis on living in the here and now. It posits that excessive focus on the past or future serves as a means to escape the present, and investing energy in the past or future diminishes present energy (Corey, 2012).

Yogacara Buddhism also provides individuals with positive psychological cues, helping them accept death as part of an endless progression of life rather than its termination. It encourages individuals to view their current life through the lens of spiritual practice, allowing them to find peace amidst the agitation caused by death anxiety, thus alleviating death anxiety.

## Conclusion

Although Buddhism is primarily considered a religion, it must exert its power not through the path of religion, but through the path of philosophical thinking and philosophical life practice. In fact, many philosophers have proposed ideas to overcome death anxiety. For example, Plato held that the soul is immortal and only the body dies (Plato, 2002). The Epicureans claimed that at the time of death, since the senses have been lost, people cannot feel death, so there is no need to fear death (Sharples, 1996). The Stoics proposed that unexpected things would bring a heavy burden to people, but if it is known that death is something that everyone will experience, then there is no need to fear death (Scherz, 2017). Hadot insisted that death is not to be feared from the perspective of universality and objectivity (Hadot, 1999). In Nussbaum (1994)'s view, what needs to be overcome is not death anxiety, but the desire for immortality. But the Buddhism are different from them. It points out that death is not a real event but just an illusion; if people cling to the concept of self and being, then this illusion will torture them, otherwise they will be able to obtain true liberation.

## References

[B1] Alkozei A. Smith R. Demers L. A. Weber M. Berryhill S. M. Killgore W. D.. (2019). Increases in emotional intelligence after an online training program are associated with better decision-making on the Iowa gambling task. Psychol. Rep. 122, 853–879. 10.1177/0033294118771705 29699472

[B2] Alvarado K. A. Templer D. I. Bresler C. Thomas-Dobson S. (1995). The relationship of religious variables to death depression and death anxiety. J. Clin. Psychol. 51, 202–204. 10.1002/1097-4679(199503)51:2<202::AID-JCLP2270510209>3.0.CO;2-M 7797643

[B3] Azam M. A. Katz J. Fashler S. R. Changoor T. Azargive S. Ritvo P.. (2015). Heart rate variability is enhanced in controls but not maladaptive perfectionists during brief mindfulness meditation following stress-induction: a stratified-randomized trial. Int. J. Psychophysiol. 98, 27–34. 10.1016/j.ijpsycho.2015.06.005 26116778

[B4] Becker E. (1973). The Denial of Death. New York: Free Press, 315.

[B5] Black D. S. Slavich G. M. (2016). Mindfulness meditation and the immune system: a systematic review of randomized controlled trials. Ann. N. Y. Acad. Sci. 1373, 13–24. 10.1111/nyas.12998 26799456 PMC4940234

[B6] Bruce A. (2007). Time (lessness): Buddhist perspectives and end-of-life. Nurs. Philos. 8, 151–157. 10.1111/j.1466-769X.2007.00310.x 17581242

[B7] CBETA (2024). Mahāvaipulya Buddhāvataṃsaka Sutra (in Chinese). R2, T10, no. 279, 102b11. Taibei: Chinese Buddhist Electronic Texts Association.

[B8] Cheung W. S. Ho S. M. (2004). The use of death metaphors to understand personal meaning of death among Hong Kong Chinese undergraduates. Death Stud. 28, 47–62. 10.1080/07481180490249265 14969278

[B9] Chiesa A. Calati R. Serretti A. (2011). Does mindfulness training improve cognitive abilities? A systematic review of neuropsychological findings. Clin. Psychol. Rev. 31, 449–464. 10.1016/j.cpr.2010.11.003 21183265

[B10] Corey G. (2012). Theory and Practice of Counseling and Psychotherapy. Belmont: Brooks/ Cole, 191–221.

[B11] Crespo-Fernández E. (2023). The death taboo: euphemism and metaphor in epitaphs from the english cemetery of Malaga, Spain. Languages 8:215. 10.3390/languages8030215

[B12] Dechesne M. Pyszczynski T. Arndt J. Ransom S. Sheldon K. M. Van Knippenberg A.. (2003). Literal and symbolic immortality: the effect of evidence of literal immortality on self-esteem striving in response to mortality salience. J. Pers. Soc. Psychol. 84:722. 10.1037/0022-3514.84.4.722 12703645

[B13] Feifel H. (1959). The Meaning of Death. New York: McGraw-Hill, 351.

[B14] Freud S. (1920). A General Introduction to Psychoanalysis. New York: Boni and Liveright, 388–402. 10.1037/10667-000

[B15] Freud S. (1981). Beyond the Pleasure Principle. S.E., 18. London: Hogarth, 3–66.

[B16] Fritsche I. Jonas E. Fankhänel T. (2008). The role of control motivation in mortality salience effects on ingroup support and defense. J. Pers. Soc. Psychol. 95, 524–554. 10.1037/a0012666 18729692

[B17] Goldenberg J. L. Arndt J. Hart J. Routledge C. (2008). Uncovering an existential barrier to breast self-exam behavior. J. Exp. Soc. Psychol. 44, 260–274. 10.1016/j.jesp.2007.05.002 19255593 PMC2276308

[B18] Greenberg J. Solomon S. Pyszczynski T. (1997). Terror management theory of self-esteem and cultural worldviews: empirical assessments and conceptual refinements. Adv. Exp. Soc. Psychol. 29, 61–139. 10.1016/S0065-2601(08)60016-7

[B19] Guoliang L. (2000). Direct Interpretation of the Ch'eng Wei-shih Lun (in Chinese). Shanghai: Fudan University Press, 804.

[B20] Hadot P. (1999). Philosophy as a Way of Life: Spiritual Exercises from Socrates to Foucault. Oxford: Blackwell Publishers Ltd., 95.

[B21] Hart J. Shaver P. R. Goldenberg J. L. (2005). Attachment, self-esteem, worldviews, and terror management: evidence for a tripartite security system. J. Pers. Soc. Psychol. 88:999. 10.1037/0022-3514.88.6.999 15982118

[B22] Hofmann S. G. Sawyer A. T. Witt A. A. Oh D. (2010). The effect of mindfulness-based therapy on anxiety and depression: a metaanalytic review. J. Consult. Clin. Psychol. 78, 169–183. 10.1037/a0018555 20350028 PMC2848393

[B23] Lehto R. Stein K. F. (2009). Death anxiety: an analysis of an evolving concept. Res. Theory Nurs. Pract. 23, 23–41. 10.1891/1541-6577.23.1.23 19418886

[B24] Lewis A. M. (2014). Terror management theory applied clinically: Implications for existential-integrative psychotherapy. Death Stud. 38, 412–417. 10.1080/07481187.2012.753557 24666148

[B25] Lusthaus D. (2002). Buddhist Phenomenology: A Philosophical Investigation of Yogacara Buddhism and the Ch'eng Wei-shih Lun. London and New York: Routledge, 275.

[B26] Maitreya (2008). Yogācāra-bhumi-śāstra (in Chinese), Vol. 1. Beijing: Religious Culture Publishing House, 15.

[B27] Menzies R. E. Zuccala M. Sharpe L. Dar-Nimrod I. (2018). The effects of psychosocial interventions on death anxiety: a meta-analysis and systematic review of randomised controlled trials. J. Anxiety Disord. 59, 64–73. 10.1016/j.janxdis.2018.09.004 30308474

[B28] Mikulincer M. Florian V. Hirschberger G. (2003). The existential function of close relationships: introducing death into the science of love. Pers. Soc. Psychol. Rev. 7, 20–40. 10.1207/S15327957PSPR0701_2 12584055

[B29] Mohammadzadeh A. Najafi M. (2020). The comparison of death anxiety, obsession, and depression between Muslim population with positive and negative religious coping. J. Relig. Health. 59, 1055–1064. 10.1007/s10943-018-0679-y 30056484

[B30] Mun-Keat C. (2000). The Fundamental Teachings of early Buddhism. Wiesbaden: Harrassowitz, 269.

[B31] Neimeyer R. A. Currier J. M. Coleman R. Tomer A. Samuel E. (2011). Confronting suffering and death at the end of life: the impact of religiosity, psychosocial factors, and life regret among hospice patients. Death Stud. 35, 777–800. 10.1080/07481187.2011.583200 24501835

[B32] Northrup D. T. (2002). Time passing: a Parse research method study. Nurs. Sci. Q. 15, 318–326. 10.1177/089431802320559245 12387230

[B33] Nussbaum M. (1994). The Therapy of Desire: Theory and Practice in Hellenistic Ethics. Princeton: Princeton University Press, 207.

[B34] Pandya A. K. Kathuria T. (2021). Death anxiety, religiosity and culture: implications for therapeutic process and future research. Religions 12:61. 10.3390/rel1201006127409075

[B35] Paranjpe A. C. (1984). Theoretical psychology: the meeting of East and West. New York: Plenum, 353. 10.1007/978-1-4613-3766-9

[B36] Plato P. (2002). Translated with Introduction and Notes by Robin Waterfield. Oxford: Oxford University Press, 100.

[B37] Pyszczynski T. Solomon S. Greenberg J. (2015). Thirty years of terror management theory: from genesis to revelation. Adv. Exp. Soc. Psychol. 52, 1–70. 10.1016/bs.aesp.2015.03.001

[B38] Scherz P. (2017). Grief, death and longing in stoic and Christian ethics. J. Relig. Ethics. 45, 7–28. 10.1111/jore.12166

[B39] Schindler S. Hilgard J. Fritsche I. Burke B. Pfattheicher S. (2023). Do salient social norms moderate mortality salience effects? A (challenging) meta-analysis of terror management studies. Pers. Soc. Psychol. Rev. 27, 195–225. 10.1177/10888683221107267 35950528 PMC10115940

[B40] Schutte N. S. Malouff J. M. (2014). A meta-analytic review of the effects of mindfulness meditation on telomerase activity. Psychoneuroendocrinology 42, 45–48. 10.1016/j.psyneuen.2013.12.017 24636500

[B41] Sharples R. W. (1996). Stoics, Epicureans and Sceptics: An Introduction to Hellenistic Philosophy. London and New York: Routledge, 94.

[B42] Vail III K. E. Soenke M. Waggoner B. (2019). “Terror management theory and religious belief,” in Handbook of terror management theory, eds. C. Routledge, and M. Vess (London: Elsevier), 259–85. 10.1016/B978-0-12-811844-3.00011-1

[B43] Van Gordon W. Shonin E. Dunn T. J. Sheffield D. Garcia-Campayo J. Griffiths M. D.. (2018). Meditation-induced near-death experiences: a 3-year longitudinal study. Mindfulness 9, 1794–1806. 10.1007/s12671-018-0922-3 30524512 PMC6244634

[B44] Xianjun X. (2023). The psychoanalytic unconscious and Buddhist unconscious (alaya-consciousness). Psychoanal. Psychother. China 6, 194–205. 10.33212/ppc.v6.2023.194

[B45] Yen C. L. (2013). It is our destiny to die: the effects of mortality salience and culture-priming on fatalism and karma belief. Int. J. Psychol. 48, 818–828. 10.1080/00207594.2012.678363 22551301

[B46] Yun P. (2009). The Sayings of Layman P'ang: A Zen Classic of China. Boston: Shambhala Publications, 130.

